# Increased production of suilysin contributes to invasive infection of the *Streptococcus suis* strain 05ZYH33

**DOI:** 10.3892/mmr.2014.2586

**Published:** 2014-09-22

**Authors:** ZHENGXIN HE, YAYA PIAN, ZHIQIANG REN, LILI BI, YUAN YUAN, YULING ZHENG, YONGQIANG JIANG, FUKUN WANG

**Affiliations:** 1 Department of Clinical Laboratory, Bethune International Peace Hospital of PLA, Shijiazhuang, Hebei 050082, P.R. China; 2 State Key Laboratory of Pathogen and Biosecurity, Institute of Microbiology and Epidemiology, Academy of Military Medical Sciences, Beijing 100071, P.R. China

**Keywords:** suilysin, invasive infection, epidemic strain, *Streptococcus suis*

## Abstract

*Streptococcus suis* serotype 2 (SS2) is widely recognized in the veterinary world as the cause of rapidly progressive and fatal sepsis in infant pigs, manifested with meningitis, polyarthritis and pneumonia. It has evolved into a highly infectious strain, and caused two large-scale outbreaks of human epidemic in China, characterized bytypical toxic-shock syndrome and invasive infection. However, the molecular basis of virulence of this emerging zoonotic pathogen is still largely unknown. The present study shows that the sequence type (ST)7 epidemic strain *S. suis* 05ZYH33 causes higher mortality, higher necrosis of polymorphonuclear neutrophils and a significantly higher damage to human umbilical vein endothelial cells compared to the non-epidemic strain *S. suis* 1940. These differences appear to associate with the enhanced secretion of suilysin (sly) by *S. suis* 05ZYH33 compared to the non-epidemic strain 1940. Inclusion of additional strains confirmed that the epidemic ST7 strains produce more sly protein (mean, 1.49 g/ml; range, 0.76–1.91 g/ml) than non-epidemic strains (mean, 0.33 g/ml; range, 0.07–0.94 g/ml), and this difference is significant (P<0.001). The nonpolar mutant strain *S. suis Δsly*, constructed from the epidemic ST7 strain *S. suis* 05ZYH33 confirmed the role of sly on the enhanced virulence of *S. suis* ST7 strains. These findings suggest that increased sly production in *S. suis* 05ZYH33 facilitates penetration to the epithelium and its survival in the bloodstream, thereby contributing to the invasive infection.

## Introduction

*Streptococcus suis* is an important emerging human threat that can cause severe systemic infection (1–3). Since the first reported cases of human infection by *S. suis* in 1968, the number of cases has significantly increased in the past few years, reaching ~1,000 cases by now, with the majority occurring in Southeast Asia. *S. suis* infections are the third most common cause of community-acquired bacterial meningitis in Hong Kong, and the leading cause of adult meningitis in Vietnam (4–5).

*The S. suis* serotype 2 (SS2) is the most common cause of the disease in humans, although serotypes 1, 4, 14 and 16 have also been reported to cause severe disease in a limited number of individuals. Most previous studies have concerned sporadic cases of *S. suis* infection, but two Chinese outbreaks (in 1998 and 2005) that involved >200 cases and 50 deaths, emphasized the importance of *S. suis* as an emerging zoonotic pathogen (6,7). The most important feature of these outbreaks was the high incidence of systemic disease, the proportionally few cases of meningitis, and the high mortality. Epidemiological surveys have indicated that all patients had a history of close contact with diseased pigs and pork-derived products, and that the emergence of the highly virulent SS2 strains played a key role in the severe outbreaks (8,9).

The current understanding of the pathogenesis of *S. suis* remains limited. Whole-genome sequencing and comparative genomic analysis (10) revealed that a DNA fragment of ~89 kb, designated as 89K, is present in the two Chinese strains (98HAH12 and 05ZYH33), and absent in the P1/7, which is a widely epidemic virulent *Streptococcus suis* serotype 2 strain. The 89K fragment was proposed to be a pathogenicity island, and genetic studies indicated that disruption of the Salk/SalR two-component system (TCS) inside the 89K considerably attenuates the virulence of the pathogen, whereas functional complementation restores virulence in infection experiments of piglets (11). A recent study (12) showed that 89K can spontaneously excise to form an extrachromosomal circular product, and the 89K excision intermediate can act as a substrate for lateral transfer to non-89K SS2 recipients via a genomic island type IV secretion system (T4SS) encoded in 89K. The authors proposed that these genetic events are important for the emergence, pathogenesis and persistence of epidemic SS2 strains. Based on sequence typing (ST), it was also suggested that ST7 strains are prevalent in China and possess a stronger capacity to stimulate T cells, naive T cells and peripheral blood mononuclear cell proliferation compared to ST1 strains (13–15). The authors of these studies proposed a two-stage hypothesis to explain streptococcal toxic shock syndrome in the Chinese outbreaks. However, both hypotheses need to be further investigated in the future.

Pore-forming toxins (PFTs) are the most prevalent virulence factors produced by disease-causing bacteria, and are required for the virulence of numerous important human pathogens, including *Staphylococcus aureus*, *S. pyogenes*, *Clostridium perfringens* and *Aeromonas hydrophilia* (16). A PFT produced by *S. suis* known as suilysin (sly) has been recognized as a virulence factor owing to its toxicity to the host epithelial cells, endothelial cells and macrophages (17–19). The sly protein belongs to a family of thiol-activated toxins and has a molecular weight of 54 kDa. It is a cholesterol-dependent cytolysin related to the streptolysin O of *S. pyogenes* and to pneumolysin of *S. pneumoniae*. Immunization to sly provides protection against lethal challenge with a serotype 2 strain in both mice and pig modelss (20,21). However, the expression profile of sly in certain *S. suis* populations and its role in causing invasive infections have not been characterized. In this study, we found that increased production of sly in the *S. suis* strain 05ZYH33 is associated with enhanced severity of *S. suis* infections and thus, may contribute to the translocation of the pathogen across the epithelial barrier. These results indicate that enhanced sly production may play an important role during *S. suis* invasive infections.

## Materials and methods

### Ethics

CD1 mice were obtained from the Experimental Animal Centre of the Academy of Military Medical Sciences (Beijing, China). Animal welfare and experimental procedures were approved by the Academy of Military Medical Sciences Animal Care and Use Committee, and were conducted in strict accordance with the Guide for the Care and Use of Laboratory Animals (National Research Council of USA, 1996). Efforts were made to minimize animal suffering and to reduce the number of animals used. For the survival experiments, the decisions were made following the relevant guidelines of the Organisation for Economic Co-operation and Development [OECD Environmental Health and Safety Publications, Series on Testing and Assessment no. 19, ENV/JM/MONO(2000)7].

### Bacterial strains and growth conditions

The bacterial strains used in this study are listed in Table I. *S. suis* was grown in Difco™ Todd-Hewitt broth (THB; Becton, Dickinson and Company, Franklin Lakes, NJ, USA) at 37°C. For antibiotic selection, 5 μg/ml of chloramphenicol (Cm) or 100 μg/ml spectinomycin (Spc) were used.

### Cell culture and lactate dehydrogenase (LDH) release assay

Human umbilical vein endothelial cells (HUVECs; Institute of Biochemistry and Cell Biology, Shanghai, China) were cultured in RPMI-1640 medium containing 10% fetal bovine serum (FBS). Cells (200 μl) were added at a density of 5×10^4^ cells/ml into 96-well culture plates. *S. suis* strains 05ZYH33 or 1940 at the mid-log phase of growth were resuspended in cell culture medium and were added to the cell culture wells at multiplicity of infection (MOI) of 100. The amount of released lactate dehydrogenase (LDH) from HUVECs was calculated with an LDH release assay kit (Promega, Madison, WI, USA) after 4 h of incubation.

### Targeted mutagenesis of the sly gene

Polymerase chain reaction (PCR) was used to generate an in-frame substitution of the *sly* gene with the *Cm* gene using a previously described method (22). Briefly, 644 and 590 bp immediately upstream and downstream of the *sly* coding sequence (SSU05_1403) were amplified with the primer pairs *sly*_upF/_upR and *sly*_downF/_downR, respectively. The *Cm* gene was amplified using the primer pair Cm_F/_R (Table II). The PCR products were cloned into the Invitrogen™ pCR2.1 plasmid (Thermo Fisher Scientific, Waltham, MA, USA), digested using restriction enzymes and subcloned into the plamsid pSET4s one by one. Allelic exchange mutagenesis in *S. suis* 05ZYH33 was performed as previously described (22) to generate the mutant *Δsly*. Allelic replacement of *sly* with *Cm* in the *S. suis* chromosome was confirmed by PCR and sequence analysis using the Phusion High-Fidelity DNA Polymerase reagents (New England Biolabs, Ipswich, MA, USA) on a ABI 3730XL machine (Applied Biosystems, Carlsbad, CA, USA).

### Enzyme-linked immunosorbent assay (ELISA) assay

The sly level in the supernatant of different *S. suis* strain cultures was determined using a double antibody sandwich ELISA assay. The antibodies used were polyclonal. Briefly, purified rabbit anti-sly IgG (1:5,000 dilution) was used to coat the wells of a microtiter plate. Purified rat anti-sly IgG (1:5,000) was used as the primary antibody, peroxidase-conjugated goat anti-rat antibody (1:5,000) was used as the secondary antibody, and K-Blue was used as the substrate (all from Santa Cruz Biotechnology, Santa Cruz, CA, USA). After 10 min of incubation at room temperature, the reaction was terminated with addition of 2 N H_2_SO_4_, and the optical density was measured at 450 nm (OD_450_) using the SpectraMax^®^ Plus384 Absorbance Microplate reader (Molecular Devices LLC, Sunnyvale, CA, USA). Purified sly protein (23) was used to establish the standard curve.

### Hemolytic activity assay

The haemolytic activity assay was performed using a previously described method (24). Briefly, the culture supernatant of *S. suis* strains was diluted 2-fold in phosphate-buffered saline (PBS). An equal amount of human red blood cells (RBCs), obtained from the Department of Hematology (Hospital 307 of Chinese People’s Liberation Army, Beijing China) and washed twice in PBS, was added to 0.5 ml of each dilution (final concentration of RBCs, 1.4%). Following incubation for 1 h at 37°C, the mixtures were sedimented by centrifugation (1,500 × g for 10 min), and the supernatants were transferred to microplates. Following correction with the controls, which lacked either haemolysin or erythrocytes, the OD_540_ of the supernatant of each dilution was measured on a SpectraMax Plus Absorbance Microplate reader.

### Reverse transcription-quantitative-PCR (RT-qPCR) analysis

The RT-qPCR analysis was performed with a *sly* gene-specific primer set (forward, ACTTACGAGCCACAAGAGATTC and reverse, GCAGCCTTAGCATCAATAACAG) on cDNA from the *S. suis* strains 05ZYH33 and 1940. The assays were carried out in triplicate using reagents of the SYBR Premix Ex Taq™ mastermix (Takara Bio, Co., Ltd., Shiga, Japan) on an Opticon 2 system (MJ Research, Waltham, MA, USA) using RNA isolated from three independent cultures for each strain. RNA was extracted using the TRIzol RNA Reagent (Invitrogen Life Technologies, Carlsbad, CA, USA) following the manufacturer’s instructions. The PCR cycling conditions were as follows: 95°C for 10 min followed by 40 cycles of 95°C for 15 sec and 60°C for 1 min. The gene encoding the 16S rRNA was used as an internal control. The Ct values were normalized to the average Ct (30) and the mean fold-changes for the target genes were calculated as described by Livak and Schmittgen (31).

### Polymorphonuclear neutrophil (PMN) phagocytosis assay

The PMN phagocytosis assay was performed as previously described (32). Human PMNs were isolated from healthy human blood as follows: blood was incubated for 40 min at room temperature in a ratio of 3:1 ratio of 6.0% Dextran to sediment erythrocytes. Ficoll, 70% percoll and blood supernatant were combined (1:1:1) and centrifuged at 400 × g for 30 min at room temperature to separate PMNs. The PMNs were aspirated in to a new cuvette, resuspended in 1640-RPMI, and enumerated by microscopy. Briefly, 1 ml of human PMNs (10^7^ cells) were mixed with an equal number of pre-opsonized *S. suis* colonies and incubated for 15 min at 37°C under continuous rotation to allow phagocytosis. When needed, different concentrations of recombinant sly protein (23) were added (50–500 ng). To quantify the intracellular bacteria, extracellular *S. suis* were killed by incubating with 100 μg/ml gentamicin and 5 g/ml penicillin for 1 h at 4°C. Subsequently, the cells were washed twice with PBS and lysed for 15 min at room temperature in 400 μl of 1% saponin. The viable bacteria were counted on an inverted IX81 microscope (Olympus, Tokyo, Japan) after plating serial dilutions of the supernatants on THB agar plates.

### Mouse infections

CD1 female mice (6-week-old) were chosen to evaluate the virulence of the *S. suis* strains 05ZYH33 and 1940. Briefly, mice (n=10 per group) were intraperitoneally injected with 10^8^ living colony forming units (CFUs) of *S. suis* strains 05ZYH33 and 1940 in 0.1 ml sterile saline. The mortality of mice infected with these bacterial strains was recorded at 4-h intervals for 32 h. To count the bacteria present in the blood, blood samples were collected from the tail vein and plated onto THB agar to accurately determine the viable bacteria at 5 h post-infection. To assess the necrosis of PMNs, we sacrificed the mice and injected 5 ml of RPMI-1640 medium containing 10% FBS into the abdominal cavities. Then, mice were surgically opened and 4-ml exudates were collected with 23-G needles. The exudates were centrifuged at 500 × g for 5 min, and cell pellets were resuspended in 100 ml of staining buffer (PBS containing 1% goat serum). The samples were stained either with fluorescein isothiocyanate (FITC)-conjugated anti-mouse anti-Ly-6G (a neutrophil marker), or with the appropriate isotype control antibody (eB149/10H5; both from eBiosciences, San Diego, CA, USA). Propidium iodide (0.5 mg/ml; BD Biosciences, San Jose, CA, USA) was used to determine the cell viability. Samples were analyzed on a FACSCalibur flow cytometer and the necrotic PMNs were quantified with the CellQuest software (both from BD Biosciences). Data were obtained from 30,000 cells for each sample.

CD1 female mice (6-week-old, ~22 g) were chosen to evaluate the virulence of the *S. suis* strain 05ZYH33 and the *Δsly* mutant. Mice (n=10, per group) were intraperitoneally injected with 0.5 ml (~5×10^8^ CFUs) of *S. suis* at the late-exponential growth phase in THB medium. The mortality of the infected mice was recorded for 50 h, and the bacteria in the blood were counted at different time-points post-infection. To histopathologically assess the severity of changes in the abdominal wall tissues, mice were surgically opened 6 h after injection. The abdominal wall tissues were fixed in 10% Formaldehyde (Sigma, St. Louis, MO, USA), sectioned and stained with hematoxylin and eosin. The slides were observed and photographed using a BX53 fluorescence microscope (Olympus, Tokyo, Japan) equipped with a DP72 CCD camera.

### Statistical analysis

Statistical analysis was performed using the SPSS 13.0 version software (SPSS Inc., Chicago, IL, USA). Statistical significance of differences was assessed with Student’s t-tests, with a p-value <0.05 considered to indicate statistically significant differences.

## Results

### The S. suis epidemic strain is more virulent than the non-epidemic strain

**Figure 1:**
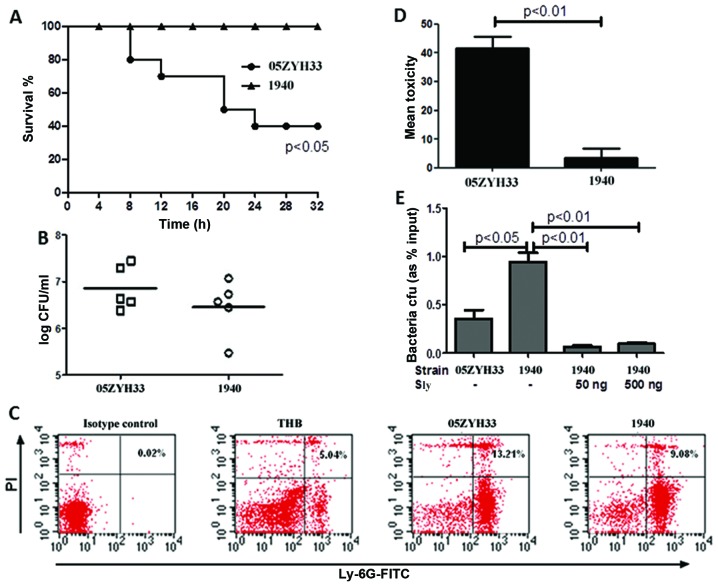
*Streptococcus suis* epidemic strain 05ZYH33 is more virulent than the non-epidemic strain 1940. (A) CD1 female mice were intraperitoneally injected with 10^8^ CFUs of living *S. suis* 05ZYH33 and 1940 in 0.1 ml sterile saline. The mortality of mice was recorded at 4-h intervals for 32 h. Cumulative results from three independent experiments, each with 10 animals per group, are shown. (B) Bacteria in the blood (n=5, per group) were counted at 5 h post-infection. (C) The necrosis of PMNs in mice infected by different bacterial strains was assayed using fluorescein isothiocyanate (FITC)-conjugated anti-mouse anti-Ly-6G and propidium iodide (PI) staining. Data represent the values obtained in four independent experiments. THB, Todd-Hewitt broth. (D) *S. suis* 05ZYH33 or 1940 (10^8^ CFUs) were incubated with human umbilical vein endothelial cells (MOI =100) for 4 h. Cytotoxicity was determined by the lactate dehydrogenase release assay. The data represent the mean ± standard deviation from three independent experiments. (E) Pre-opsonised *S. suis* 05ZYH33 or 1940 (10^7^ CFUs) were incubated with purified human polymorphonuclear neutrophils (MOI =1) in the presence or absence of recombinant sly protein for 15 min. The number of bacteria phagocytized by the PMNs was counted.

The *S. suis* epidemic strains are expected to show high virulence, and need to resist host defence mechanisms and be able to invade the epithelial or the endothelial barrier in order to cause successful infection. To evaluate the potential differences in virulence between *S. suis* epidemic and non-epidemic strains, CD1 female mice were intraperitoneally injected with 10^8^ CFUs of the *S. suis* strains 05ZYH33 (epidemic, ST7) or 1940 (non-epidemic) in 0.1 ml sterile saline. The strain 05ZYH33 caused significantly higher mortality (p<0.05) than the 1940 strain (Fig. 1A). The bacterial concentration in the blood of the infected mice also appeared different at 5 h post-infection, although the difference was not statistically significant (Fig. 1B). In addition, *S. suis* 05ZYH33 caused higher necrosis of PMNs (Fig. 1C), the major cell type mediating acute inflammatory responses to bacterial infections, compared to *S. suis* 1940 (13.21±3.11 vs. 9.08±2.56%). Next, we used HUVECs to test the ability of the two *S. suis* strains to damage the cells. The results showed that 05ZYH33 has a higher cytotoxicity than 1940, as determined by the LDH assay (Fig. 1D). Taken together, these data demonstrate that the *S. suis* epidemic strain 05ZYH33 is more virulent than the non-epidemic strain 1940.

Next, we investigated whether the sly protein may contribute to the enhanced virulence of the *S. suis* epidemic strain. Pre-opsonised *S. suis* 05ZYH33 or 1940 were incubated with purified human PMNs (MOI =1) for 15 min in the presence or absence of the sly protein, and the bacteria phagocytized by PMNs were counted. This assay showed that PMNs phagocytized the *S. suis* 1940 strain at higher rates compared to the 05ZYH33 strain, (Fig. 1E) while adding sly protein significantly decreased the phagocytosis of *S. suis* 1940 by the PMNs.

### The S. suis epidemic strain 05ZYH33 shows increased production of sly compared to the non-epidemic strains

**Figure 2:**
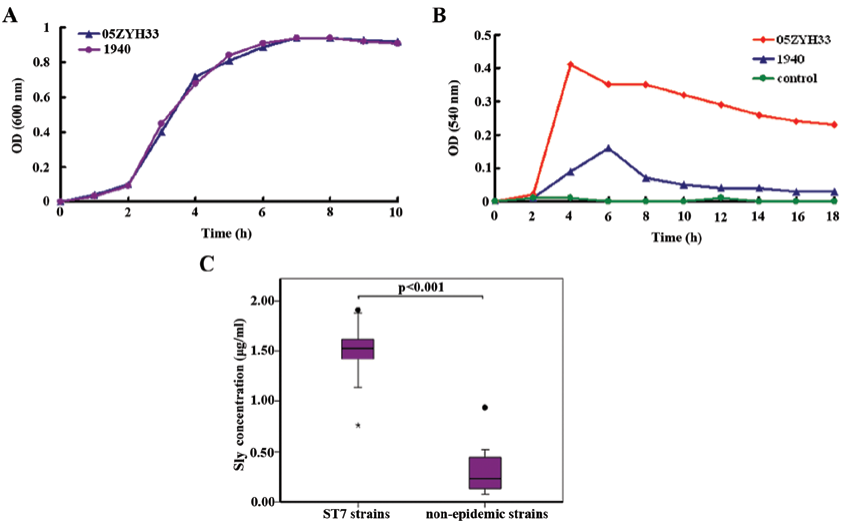
*Streptococcus suis* ST7 epidemic strains show increased production of suilysin (sly) compared to non-epidemic strains. *S. suis* 05ZYH33 and 1940 (A) growth curves and (B) relative hemolytic activities, assessed in the supernatants collected at different time-points. (C) Sly protein concentration, determined by an enzyme-linked immunosorbent assay, performed on 11 ST7 strains and 8 non-epidemic strains listed in Table I.

Since the sly protein displays hemolytic activity, we then compared the relative amounts of sly produced by the *S. suis* epidemic strain 05ZYH33 and the non-epidemic strain 1940. Although both strains showed the same growth rate (Fig. 2A), the supernatant of *S. suis* 05ZYH33 displayed higher hemolytic activity than that of *S. suis* 1940 (Fig. 2B). This phenotypic difference was further confirmed by qRT-PCR, which indicated a difference in *sly* expression between the two *S. suis* strains; a 6.3-fold difference at the mRNA transcription level of *sly* was observed between the two strains (data not shown).

In order to confirm the hypothesis that the *S. suis* ST7 epidemic strains may have increased production of sly compared to the non-epidemic strains, 11 *S. suis* ST7 epidemic strains and eight non-epidemic strains were collected (Table I), and the amount of sly was determined in the supernatant at 7 h of culture, using a sandwich ELISA assay (Fig. 2C). The epidemic ST7 strains produced more sly (mean, 1.49 g/ml; range, 0.76–1.91 g/ml) than the non-epidemic strains (mean, 0.33 g/ml; range, 0.07–0.94 g/ml). Moreover, the difference between the two groups was significant (p<0.001).

### Sly is associated with enhanced severity of S. suis infections

**Figure 3:**
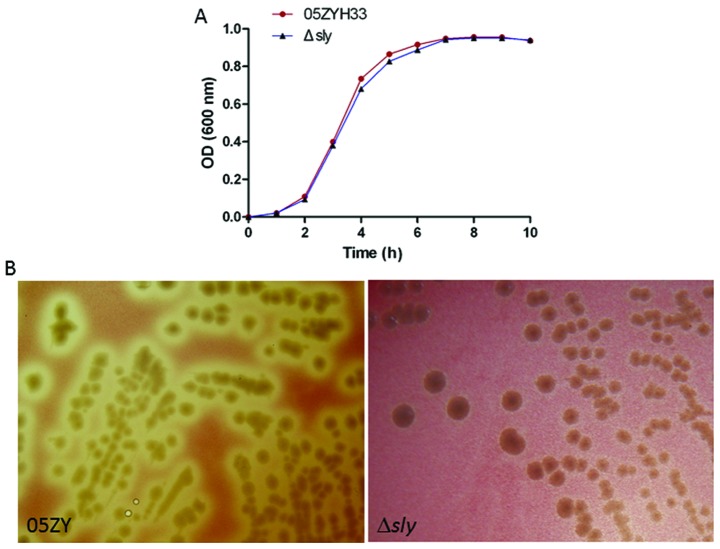
The mutant *Δsly* strain shows (A) similar growth rates compared to the wild-type strain *S. suis* 05ZYH33, but (B) reduced hemolytic activity.

**Figure 4:**
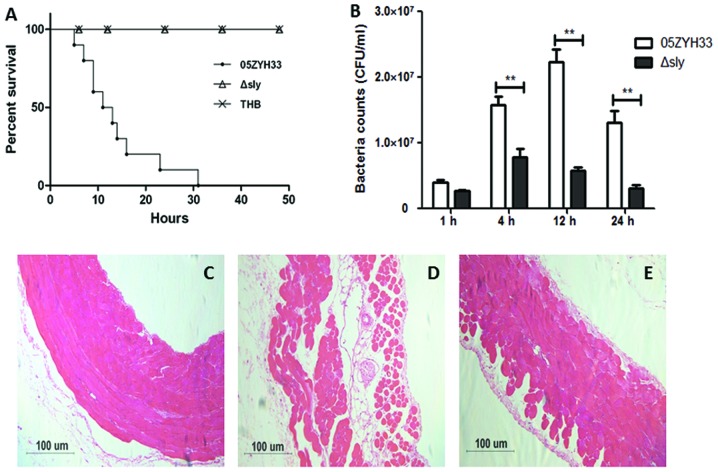
Suilysin is associated with the enhanced severity of *S. suis* 05ZYH33 infections. CD1 mice (6-week-old, female, ~22 g) were intraperitoneally injected with 0.5 ml (~5×10^8^ CFUs) *S. suis* at the late-exponential growth phase, supplemented with Todd-Hewitt broth (THB). (A) The mortality of the infected mice, recorded for 50 h. (B) The bacterial count in the blood at different time-points post-infection. The abdominal wall tissues were histopathologically examined 6 h after injection of (C) THB, (D) 05ZYH33, and (E) *Δsly*, using hematoxylin and eosin staining. Images are representative of two independent experiments.

To further explore the roles of sly in invasive infection of the 05ZYH33 strain, the *sly* gene was in-frame substituted by the *Cm* gene in the *S. suis* 05ZYH33 strain, to generate the nonpolar mutant *Δsly*. The mutant *Δsly* strain showed reduced hemolytic activity, but similar growth rates compared to the wild-type strain *S. suis* 05ZYH33 (Fig. 3). We further investigated the virulence of this mutant. CD1 mice (6-week-old female, ~22 g) were infected by intraperitoneal injection with 0.5 ml (~5×10^8^ CFUs) *S. suis* at the late-exponential growth phase in THB. The mortality of the infected mice was recorded for 50 h. No mice died in the *Δsly* or the THB control group, while the mice infected with *S. suis* 05ZYH33 all died within 32 h post-infection. The bacterial concentration in the blood was evaluated at different time-points post-infection (Fig. 4A). The parental 05ZYH33 strain survived/multiplied in the blood at higher rates than *Δsly* at 4, 12 and 24 h (Fig. 4B). Histopathological examination of the abdominal wall tissues was also performed 6 h after the infection. The abdominal epithelial cells of mice infected by *S. suis* 05ZYH33 lost their normal morphological features and the integrity of the tight junctions, while the epithelial cells infected by *S. suis Δsly* showed a few changes (Fig. 4C–E). Taken together, these data demonstrate that sly is associated with enhanced severity of *S. suis* 05ZYH33 infections.

## Discussion

*S. suis* is a swine pathogen responsible for a number of infections, including meningitis, endocarditis and septicemiae, and is also an important zoonotic agent. The two outbreaks that occurred in China in 1998 and 2005 affected the world perspective regarding the threat that this pathogen represents for humans. Subsequent investigations on *S. suis* at the molecular and the genomic level confirmed that the emergence of the highly virulent SS2 strains, or epidemic ST7 strains, played a key role in these severe outbreaks (13). In the present study, we sought to determine the capacity of the *S. suis* protein sly to enhance the virulence of the epidemic ST7 strain 05ZYH33.

A previous study showed that *S. suis* ST7 strains express the proposed virulence markers muramidase-released protein (MRP), extracellular protein factor (EF), and sly (15). However to date, there is no study that specifically focused on how the differences in the expression abundance of bacterial virulence factors may affect the severity of *S. suis* infections. We hereby showed, using an animal model, that the *S. suis* ST7 epidemic strain 05ZYH33 causes higher mortality, higher necrosis of PMNs and significantly higher damage to HUVECs than the *S. suis* non-epidemic strain 1940. In agreement with these results, epidemic ST7 strains produced more sly than non-epidemic strains. This significant difference in sly secretion may be of outmost importance in *S. suis* 05ZYH33 infections. Phagocytosis is the first line of cellular defense against acute infectious diseases. A clinical association with invasive human infections implies that bacteria can survive the innate host defense responses raised in order to clear the bacteria from the bloodstream and the tissues. In other pathogenic streptococcal infections, hemolysins have been shown to be involved in bacteria-phagocyte interactions. For example, in Group B *Streptococcus* (GBS) infections, Liu *et al* (32) elegantly demonstrated the multi-factorial roles of the pore-forming toxin β-hemolysin/cytolysin (βH/C) in thwarting the immune phagocytic defenses. In the present study, we demonstrated that sly mediates inhibition of the phagocytic uptake by human PMNs *in vitro*. Moreover, we demonstrated that the *S. suis* strain with enhanced sly expression causes enhanced PMN necrosis in an *in vivo* mouse model. Taken together, these findings suggest that the sly-mediated inhibition of phagocytosis and cytolysis is directly associated with a mechanism impairing the function and viability of PMNs, and thus contributing to the enhanced survival of epidemic *S. suis* strains.

The ability of sly to enhance the severity of *S. suis* infection was confirmed using a mutant of *S. suis* 05ZYH33 deficient in *sly* expression. The survival rate of mice challenged by the *Δsly* strain was significantly increased, and the bacterial count in the blood decreased compared to the parental strain 05ZYH33. These data strongly support the contribution of sly in enhancing the severity of *S. suis* ST7 strain infections, and are consistent with the previous study of Allen *et al* (33).

In our intraperitoneal infection animal model, *S. suis* needs to successfully cross the abdominal epithelial barrier and survive in the bloodstream in order to cause invasive infection. Among the potential strategies that can allow this are: transcellular transport by passive or adhesion-induced transcytosis, paracellular passage through opened tight junctions, disruption of the barrier due to a direct cytotoxic effect, leukocyte-facilitated transport by infected phagocytes (34). Histopathological examination showed that the mice infected by *S. suis* 05ZYH33 lost their abdominal epithelial cell morphological features and the integrity of the tight junctions compared to mice infected by the mutant *Δsly*. The *S. pneumoniae* toxin pneumolysin recognizes Toll-like receptor (TLR)4 on dendritic cells (35), and *S. pneumoniae* and *H. influenzae* were shown to exploit TLR-dependent downregulation of claudins 7 and 10, tight junction key components for the maintenance of the epithelial barrier integrity(36), to facilitate translocation across the epithelium (37). However, *in vivo* studies carried out in our laboratory indicate that the *S. suis* sly protein contributes to the release of inflammatory cytokines in a TLR4-independent manner (data not shown). Whether activation of TLR4 by sly can facilitate the *S. suis* translocation across the epithelium needs to be further studied.

In conclusion, we have shown that differences in sly production are linked to differences in *S. suis* virulence. The pathogenic *S. suis* ST7 strain 05ZYH33 appeared to have an increased ability to subvert host clearance mechanisms, which may allow its survival and dissemination into the bloodstream. The cytotoxic effects of sly may inhibit the uptake of microorganisms by the host phagocytes and can directly cause epithelial cell damage, enhancing the microbial spread into deeper tissues. Thus, in a murine model, sly contributes to increased bacterial load and death. Further understanding of the precise molecular mechanisms underlying the sly-mediated pathogenicity may provide potential therapeutic targets for a better control of *S. suis* infections.

## Figures and Tables

**Table I:** Bacterial strains and plasmids used in this study.

Material	Description	Area (year)-Origin	Status	Source/Ref.
*Streptococcus suis strains*				
*Δsly*	In frame deletion of *sly* in 05ZYH33	Beijing, China (2011) - Our lab	Mutant	This study
1330	Sly^−^89K^−^	Canada-HPL	Avirulent	(25)
05ZYH33	Sly^+^89K^+^	Sichuan, China (2005)-HP	Epidemic	This study
98001	Sly^+^89K^+^	Jiangsu, China (1998)-HP	Epidemic	This study
99001	Sly^+^89K^+^	Jiangsu, China (1998)-HP	Epidemic	This study
98003	Sly^+^89K^+^	Jiangsu, China (1998)-HP	Epidemic	This study
98005	Sly^+^89K^+^	Jiangsu, China (1998)-HP	Epidemic	This study
98012	Sly^+^89K^+^	Jiangsu, China (1998)-HP	Epidemic	This study
98015	Sly^+^89K^+^	Jiangsu, China (1998)-HP	Epidemic	This study
98242	Sly^+^89K^+^	Jiangsu, China (1998)-HP	Epidemic	This study
4	Sly^+^89K^+^	Sichuan, China (2005)-DP	Epidemic	This study
5	Sly^+^89K^+^	Sichuan, China (2005)-DP	Epidemic	This study
SUN	Sly^+^89K^+^	Jiangsu, China (2006)-HP	Epidemic	This study
606	Sly^+^89K^−^	China (1980)-DP	Non-epidemic	This study
607	Sly^+^89K^−^	Japan-DP	Non-epidemic	This study
1940	Sly^+^89K^−^	China (1980)-DP	Non-epidemic	This study
1941	Sly^+^89K^−^	China (1980)-DP	Non-epidemic	This study
NJ	Sly^+^89K^−^	Jiangsu, China-DP	Non-epidemic	This study
4005	Sly^+^89K^−^	The Netherlands-DP	Non-epidemic	(26)
s735	Sly^+^89K^−^	The Netherlands-DP	Non-epidemic	(27)
T15	Sly^+^89K^−^	Europe-HPL	Non-epidemic	(28)
Plasmids				
pCR2.1	na	na	na	Invitrogen™
pSET4s	na	na	na	(29)

Sly, suilysin; 89K, 89 kb pathogenicity island; HPL, healthy piglets; HP, human patients; DP, diseased piglets; na, not applicable.

**Table II:** Oligonucleotide primer sequences used in this study.

Primer	Sequence (5′-3′)	Restriction enzyme
Sly_upF	GGGGGAAGCTTCTAGTCGGGGGAGTTTTTGTG	*Hin*dIII
Sly_upR	GGGGGGGTCGACTTAATATCTTGTTTGGAATCTG	*Sal*I
Cm_F	GCGGTCGACTAATTCGATGGGTTCCGAGG	*Sal*I
Cm_R	CGCGGATCCCACCGAACTAGAGCTTGATG	*Bam*HI
Sly_downF	GGGGGGATCCCATGGGAGTGGTGGAGAACAGT	*Bam*HI
Sly_downR	GGGGGGGGAATTCTTGGCCCGAATACCGACAG	*Eco*RI

Sly, suilysin; up, upstream; down, downstream; F, forward; R, reverse; Cm, chloramphenicol; underlined, restriction sites.
